# Cancer in a drop: Liquid biopsy highlights from San Antonio Breast Cancer Symposium (SABCS) 2024

**DOI:** 10.1016/j.jlb.2025.100286

**Published:** 2025-01-09

**Authors:** Letizia Pontolillo, Mohamed A. Gouda, Konstantinos Venetis, Eleonora Nicolò, Carolina Reduzzi

**Affiliations:** a Division of Hematology-Oncology, Weill Cornell Medicine, 420 E 70th St, LH 204, New York, NY, 10021, USA; b Department of Translational Medicine and Surgery, Università Cattolica del Sacro Cuore, Largo Francesco Vito, 00168, Rome, Italy; c Department of Investigational Cancer Therapeutics, The University of Texas MD Anderson Cancer Center, 1515 Holcombe Boulevard, Unit 455, Houston, TX, USA; d Division of Pathology, IEO European Institute of Oncology IRCCS, 20141, Milan, Italy; e International Society of Liquid Biopsy (ISLB) Young Committee, Spain

**Keywords:** Breast cancer, Liquid biopsy, ctDNA, CTCs, SABCS 2024

## 1 Introduction

The San Antonio Breast Cancer Symposium (SABCS) serves as an annual comprehensive educational and scientific platform, covering basic, translational, and clinical sciences with the shared goal of advancing care for patients with breast cancer (BC). This editorial, authored on the behalf of the International Society of Liquid Biopsy (ISLB) Young Committee, highlights key presentations centered on liquid biopsy—a topic of growing interest in the field of breast cancer. Notably, this year, over 100 abstracts focusing on circulating biomarkers, primarily circulating tumor DNA (ctDNA), were presented in SABCS. Here, we report a selection of abstracts, organized by application in four main areas: i) minimal residual disease (MRD) monitoring, ii) identification of predictive biomarkers, iii) response evaluation, and iv) challenging settings.

## 2 Minimal residual disease monitoring

Several presentations at the conference highlighted the value of MRD detection, exploring tumor-informed and tumor-agnostic approaches, as well as combinatorial strategies.

The results from the ZEST trial [1], the first phase III study of MRD-guided therapy in BC, were presented. This trial was designed to evaluate niraparib to prevent recurrence in ctDNA-positive patients using the Signatera™ tumor-informed assay. Prescreening over 2700 stage I-III patients with triple negative breast cancer (TNBC) or BRCA-mutated human epidermal growth factor receptor 2 (HER2)-negative BC, only 8 % were ctDNA-positive. However, nearly half of these patients already had metastatic disease linked to higher ctDNA level, leading to insufficient accrual and early trial termination. Nevertheless, in the 40 patients with positive MRD who were randomized to niraparib or placebo, recurrence-free survival (RFS) was numerically longer with niraparib (11.4 *vs* 5.4 months with placebo), suggesting potential benefits of treatment escalation in patients with ctDNA detection, although lack of statistical significance requires further validation. Moreover, an earlier approach integrating ctDNA thresholds with tumor burden models for risk stratification for TNBC should be investigated [2].

Underscoring the rationale for ctDNA-informed therapeutics strategies, in a real-world study, the Signatera™ assay identified actionable mutations in 45 % of ctDNA-positive patients with hormone receptor positive (HR-positive)/HER2-negative breast cancer, with Phosphatidylinositol-4,5-bisphosphate 3-kinase catalytic subunit alpha (*PIK3CA*) mutations (35 %) linked to worse RFS [3]. To better define the risk of recurrence, the SURMOUNT study [4] demonstrated complementary insights from combining disseminated tumor cells (DTCs) and ctDNA tumor-informed testing (RaDaR assay), covering dormancy and active shedding phases and offering an extended window for early intervention.

The integration of advanced technologies to analyze different circulating analytes, such as circulating tumor cells (CTCs) and ctDNA, has demonstrated clinical validity even in rare and aggressive diseases like inflammatory breast cancer (IBC). In stage III IBC patients, these approaches have enabled the detection of disease recurrence approximately six months prior to radiographic confirmation [5].

From a tumor-agnostic perspective, new studies expanded MRD applications. Pai et al. described the feasibility of Guardant Reveal tissue-free assays in 2000 patients in a large, unselected BC cohort, suggesting a potential clinical practice attitude of monitoring for stage III TNBC and HR+/HER2-negative BC [6].

Structural variants (SVs)-based assays gained prominence. The ALIENOR study [7] validated SVs-based digital polymerase chain reaction (dPCR) for relapse prediction after neoadjuvant treatment in patients that did not achieve pathological complete response (pCR) with lead times up to 61 months, offering a promising window for early intervention. Nevertheless, the clearance obtained on adjuvant therapy, suggested the possibility of a micrometastatic disease control. Elliott et al. supported [8] the feasibility of personalized SVs panels with exceptional sensitivity, while Acevedo et al. highlighted the development of new second-generation tumor informed assays achieving detection limits below 5 part per million (ppm) [9].

## 3 Predictive biomarkers

The potential to detect predictive biomarkers through liquid biopsy was explored in several abstracts, including biomarker analyses from prospective trial.

In the DESTINY-Breast03 trial [10], the genomic landscape at treatment-baseline and progression was investigated using the GuardantINFINITY assay, showing no genomic impact on the response to Trastuzumab-Deruxtecan (T-DXd) compared to Trastuzumab emtansine (T-DM1), although a trend towards reduced efficacy was observed for T-DM1 in case of low HER2 plasma median copy number and Phosphatidylinositol 3-kinase (*PI3K*) mutations. Interestingly, Topoisomerase I (*TOP1*) mutations were detected at progression only in the T-DXd arm, suggesting a possible mechanism of resistance to the drug payload, rather than the antibody. In the context of cyclin-dependent kinase 4/6 (CDK4/6) inhibitor beyond progression (BP), in the PACE study [11], the DNADX assay allowed for the prognostic classification of patients into five subgroups: luminal-high, proliferative, basal-related, copy-number aberration (CNA)-flat, and tumor fraction (TF)-low (TF < 3 %). This study showed that the luminal-high subgroups is the one that might derive greater benefit from continuation of CDK4/6 inhibitor BP. In the same setting, looking into single gene mutations, Retinoblastoma 1 (*RB1)* and Phosphatase and tensin homolog (*PTEN)* were related to diminished efficacy of abemaciclib in the postMONARCH trial [12], even though the limited incidence required further investigations. Finally, an additional analysis of the EMERALD trial demonstrated the efficacy of elacestrant for HR-positive/HER2-negative metastatic breast cancer (mBC), regardless of high or low Estrogen Receptor 1 *(ESR1)* variant allele frequency (VAF) or the specific *ESR1* mutation variant [13].

The TAKTIC phase Ib trial [14] highlighted an enhanced response to Protein kinase B (*AKT*) inhibitor -based therapy (ipatasertib + fulvestrant + palbociclib) in patients with alterations in the PI3K/AKT/PTEN pathway whereas Fibroblast Growth Factor Receptor 1 (*FGFR1)* alterations were linked to worse outcome; given the small cohort, cautious interpretation is warranted. Lastly, the predictive value of CTCs was evaluated in the phase II/III NRG-BR002 study [15], which randomized patients with oligometastatic BC to standard systemic therapy (SOC) with or without ablation (surgery or radiation) of all visible metastases. While ablation did not improve progression-free survival (PFS) in the overall population, patients with low CTCs levels prior to treatment demonstrated improved outcomes with ablation. In a multivariable analysis adjusting for the number of metastases, the interaction between treatment arm and CTCs levels maintained the statistical significance.

## 4 Response evaluation

Liquid biopsy is emerging as a valuable tool for evaluating treatment responses, as highlighted in various presentations. Dynamic changes in liquid biopsy components, such as ctDNA and CTCs, have been linked to clinical outcomes in diverse settings.

The IMAGE II study analyzed longitudinal ctDNA profiles in patients with mBC undergoing FoundationOne® Liquid CDx testing before treatment initiation. A 90 % reduction in TF within 1–2 weeks from treatment initiation correlated with clinical response at first restaging, providing early insights into treatment efficacy, while changes in maximum VAF lacked statistical significance [16]. Similarly, Foffano et al. investigated ctDNA VAF as a marker of disease progression in patients with mBC tested with Guardant360®, demonstrating that a 0 % cut-off improved sensitivity in detecting progression [17].

Several phase I/II trials have demonstrated the feasibility of monitoring response to novel treatments using liquid biopsy markers. For instance, ctDNA clearance or *ESR1* allele frequency changes tracked responses to a Histone lysine acetyltransferase (KAT6) inhibitor [18], while decreases in CTCs indicated efficacy for a chimeric estrogen receptor degrader [19]. Larger studies are needed to confirm these findings.

In early-stage BC, Carelton et al. used the Signatera™ assay to track ctDNA in elderly HR+/HER2-negative patients who received primary endocrine therapy (ET) in a prospective study exploring surgical de-escalation as a feasible approach; and supporting its non-inferiority to standard surgical management [20]. Patients with a pre-treatment negative ctDNA or positive ctDNA with clearance after ET did not have progression, while patients with positive ctDNA without further clearance had disease progression. The PELOPS phase II trial validated the new generation Guardant Infinity assay for monitoring neoadjuvant palbociclib + ET response, demonstrating its prognostic value in recurrence prediction [21]. Similarly, Liang et al. showed that decreased ctDNA positivity after neoadjuvant treatment with dalpiciclib and targeted agents in patients with HR-positive/HER2-positive BC correlated with better outcomes, while lack of ctDNA clearance predicted lower pCR rates [22].

## 5 Challenging settings

Some studies highlighted the role of liquid biopsy in advancing our understanding of the unique biology underlying BC subtypes, such as lobular (ILC) and IBC. In ILC, ctDNA analyses revealed a distinct mutational profile and focal chromosomal aberrations compared to non-special type (NST) BC [23,24]. Moreover, the analysis of CTC clusters, considered the main seed of metastasis, showed that ILC clusters are more abundant, smaller, and more frequently associated with white blood cells, suggesting a unique biology influenced by impaired cell-cell adhesion of ILC and the tumor microenvironment [25]. A promising role emerged for novel analytes like exosomes; one study identified IBC-specific protein signatures in exosomes [26], potentially linked to the immune-suppressive tumor microenvironment that characterizes IBC. Another challenging scenario is leptomeningeal metastasis [27], where genomic profiling of CTCs in cerebrospinal fluid (CSF) has revealed a high level of clonality and distinct aberration patterns compared to extracranial disease. Notably, some mutations in resistance-related and tumor driver genes were detected exclusively in CSF-CTCs, underscoring their potential role in guiding therapy selection.

Finally, it is worth mentioning the role of liquid biopsy in addressing disparities in BC care and in the incidental detection of germline mutations. Podany et al. [28], using a multi-institutional ctDNA database, reported that GATA Binding Protein 3 (*GATA3)* Single Nucleotide Variants (SNVs), Cyclin D1 (*CCND1)* CNVs, and cell cycle pathway alterations were more common in Black patients with de novo HR-positive/HER2-negative mBC than in White patients, possibly contributing to the more aggressive tumor biology and poorer outcomes observed in this group. Data provided from the same multi-institutional consortium [29], suggested that ctDNA analysis can detect germline mutations with VAF lower than expected, which has important implications for patient treatment and screening strategies.

## 6 Conclusion

The 2024 SABCS showcased the expanding role of liquid biopsy in advancing precision oncology for BC, with MRD emerging as a key area of interest. While evidence highlights its potential for early detection of recurrence and risk stratification, validating its clinical utility and impact on patient outcomes remains a priority. Future efforts should focus on developing standardized methods, incorporating liquid biopsy into routine clinical practice to identify predictive biomarkers and track treatment response, and reducing disparities to ensure equal access and benefits for all patients.

## Author's contribution

All authors have contributed equally to the conception, drafting, and revision of this editorial.

## Ethical approval/patient consent

Not required.

## Ethical approval

No ethical approvals or patient consent were necessary for the study.

## Funding

The authors have not declared a specific grant for this review from any funding agency in the public, commercial or not-for-profit sectors.

## Declaration of competing interest

The authors declare the following financial interests/personal relationships which may be considered as potential competing interests: Letizia Pontolillo reports a relationship with Pfizer that includes: travel reimbursement. Letizia Pontolillo reports a relationship with Eli Lilly that includes: travel reimbursement. Letizia Pontolillo reports a relationship with Gilead Sciences Inc that includes: travel reimbursement. Mohamed A Gouda reports a relationship with Caris that includes: consulting or advisory. Carolina Reduzzi reports a relationship with 10.13039/100000043American Association for Cancer Research that includes: funding grants. Authors declare to be members of the Young Committee of the International Society of Liquid Biopsy. If there are other authors, they declare that they have no known competing financial interests or personal relationships that could have appeared to influence the work reported in this paper.
